# Large Language Models in Worldwide Medical Exams: Platform Development and Comprehensive Analysis

**DOI:** 10.2196/66114

**Published:** 2024-12-27

**Authors:** Hui Zong, Rongrong Wu, Jiaxue Cha, Jiao Wang, Erman Wu, Jiakun Li, Yi Zhou, Chi Zhang, Weizhe Feng, Bairong Shen

**Affiliations:** 1 Joint Laboratory of Artificial Intelligence for Critical Care Medicine, Department of Critical Care Medicine and Institutes for Systems Genetics Frontiers Science Center for Disease-related Molecular Network, West China Hospital Sichuan University Chengdu China; 2 Shanghai Key Laboratory of Signaling and Disease Research School of Life Sciences and Technology Tongji University Shanghai China; 3 Department of Neurosurgery First Affiliated Hospital of Xinjiang Medical University Urumqi China; 4 Department of Urology West China Hospital Sichuan University Chengdu China; 5 West China Tianfu Hospital Sichuan University Chengdu China

**Keywords:** large language models, LLMs, generative pretrained transformer, ChatGPT, medical exam, medical education, artifical intelligence, AI

## Abstract

**Background:**

Large language models (LLMs) are increasingly integrated into medical education, with transformative potential for learning and assessment. However, their performance across diverse medical exams globally has remained underexplored.

**Objective:**

This study aims to introduce MedExamLLM, a comprehensive platform designed to systematically evaluate the performance of LLMs on medical exams worldwide. Specifically, the platform seeks to (1) compile and curate performance data for diverse LLMs on worldwide medical exams; (2) analyze trends and disparities in LLM capabilities across geographic regions, languages, and contexts; and (3) provide a resource for researchers, educators, and developers to explore and advance the integration of artificial intelligence in medical education.

**Methods:**

A systematic search was conducted on April 25, 2024, in the PubMed database to identify relevant publications. Inclusion criteria encompassed peer-reviewed, English-language, original research articles that evaluated at least one LLM on medical exams. Exclusion criteria included review articles, non-English publications, preprints, and studies without relevant data on LLM performance. The screening process for candidate publications was independently conducted by 2 researchers to ensure accuracy and reliability. Data, including exam information, data process information, model performance, data availability, and references, were manually curated, standardized, and organized. These curated data were integrated into the MedExamLLM platform, enabling its functionality to visualize and analyze LLM performance across geographic, linguistic, and exam characteristics. The web platform was developed with a focus on accessibility, interactivity, and scalability to support continuous data updates and user engagement.

**Results:**

A total of 193 articles were included for final analysis. MedExamLLM comprised information for 16 LLMs on 198 medical exams conducted in 28 countries across 15 languages from the year 2009 to the year 2023. The United States accounted for the highest number of medical exams and related publications, with English being the dominant language used in these exams. The Generative Pretrained Transformer (GPT) series models, especially GPT-4, demonstrated superior performance, achieving pass rates significantly higher than other LLMs. The analysis revealed significant variability in the capabilities of LLMs across different geographic and linguistic contexts.

**Conclusions:**

MedExamLLM is an open-source, freely accessible, and publicly available online platform providing comprehensive performance evaluation information and evidence knowledge about LLMs on medical exams around the world. The MedExamLLM platform serves as a valuable resource for educators, researchers, and developers in the fields of clinical medicine and artificial intelligence. By synthesizing evidence on LLM capabilities, the platform provides valuable insights to support the integration of artificial intelligence into medical education. Limitations include potential biases in the data source and the exclusion of non-English literature. Future research should address these gaps and explore methods to enhance LLM performance in diverse contexts.

## Introduction

Generative pretrained transformers (GPTs) and large language models (LLMs) have revolutionized the field of natural language processing. These models, supported by advanced techniques such as deep learning and attention mechanisms, have demonstrated remarkable capabilities for understanding and generating human-like text. ChatGPT, developed by OpenAI, has garnered widespread attention for its ability to engage in coherent and context-aware conversations [1]. Additionally, there are other models such as Llama [2], Bard [3], and PaLM [4]. These LLMs have had a significant impact in the fields of biomedicine and health care [5-8], particularly in the context of medical education [9-11].

Medical exams, such as licensing and certification tests for health care professionals, play a vital role in improving patient safety and practitioner competence and hold immense significance in medical education and the health care system [12]. These rigorous exams require a deep understanding of medical knowledge, advanced clinical reasoning skills, and contextual understanding of real-world patient scenarios. Medical exams serve as a crucial step in the process of becoming a certified and licensed medical professional [13]. The exams assess the medical knowledge and clinical skills of health care professionals, including physicians, surgeons, nurses, pharmacists, and students [14-17]. The medical exams cover a wide range of medical topics, including basic sciences (eg, anatomy, biochemistry, physiology), clinical medicine (eg, internal medicine, pediatrics, surgery, psychiatry, obstetrics and gynecology), and even specialized areas. These medical exams generally take the form of multiple-choice questions or short-answer questions. Preparing for the medical exam requires extensive studying of medical textbooks, clinical guidelines, and relevant publications. Various resources, such as question banks, practice exams, and online learning platforms, can be used to prepare for the medical exam.

LLMs have demonstrated considerable potential in medical education, as well as considerable capabilities for medical exams worldwide [18,19]. A large number of studies have been conducted to evaluate the performance of LLMs in medical exams across different countries and languages [20,21]. For example, ChatGPT has shown promising results for the United States Medical Licensing Exam, which consists of 3 steps: Step 1, Step 2CK, and Step 3 [22-24]. ChatGPT has also exhibited great potential for the Chinese National Medical Licensing Examination, Chinese National Pharmacist Licensing Examination, and Chinese National Nurse Licensing Examination during a 5-year evaluation study [17]. Other models like GPT-4, Bing, and GPT-3.5-Turbo have demonstrated their capabilities for the German Medical State Examinations [25], Japanese National Medical Licensing Examination [26], Australian Medical Council Licensing Examination [27], and Korean National Licensing Examination for Korean Medicine Doctors [28]. The performance evaluation of LLMs in these medical exams necessitates not only the understanding of complex medical concepts but also translational application for solving intricate clinical problems.

It is the first time that such a substantial number of studies have delved into the potential and capability of artificial intelligence models for medical exams worldwide. These studies not only present evaluation results and research evidence but also offer valuable insights for advancing LLMs in biomedicine and health care [29]. Furthermore, these studies have facilitated the integration of artificial intelligence into medical education. 

Despite the growing interest in LLM performance on medical exams, existing studies remain fragmented, with data scattered across diverse sources and lacking standardization. This fragmentation creates a critical gap in understanding the broader trends and disparities in LLM capabilities across geographic, linguistic, and contextual boundaries. Although systematic reviews synthesize evidence, they do not provide an interactive, centralized system to continuously analyze, update, and compare LLM performance on medical exams. Moreover, current evidence often varies in quality, and not all studies are readily accessible to researchers and educators. To address these gaps, we proposed developing a comprehensive platform that provides a centralized system for collecting, analyzing, and comparing evidence-based knowledge regarding the performance of various LLMs across a wide range of medical exams around the world.

In this study, we introduce MedExamLLM, a platform specifically designed for benchmarking LLMs for medical exams around the world. The main contributions of our study lie in 3 key aspects: (1) We present MedExamLLM, an open-source, freely accessible, and publicly available platform, which serves as a crucial resource by providing performance evaluation information and evidence-based knowledge of the comprehensive capability of LLMs on medical exams worldwide; (2) MedExamLLM compiles data on 16 LLMs across 198 medical exams conducted in 28 countries, covering 15 languages, and spanning from the year 2009 to the year 2023; (3) MedExamLLM enables information retrieval, in-depth analysis, and performance comparison, which can facilitate translational research of artificial intelligence technologies in health care and medical education.

**Figure 1:**
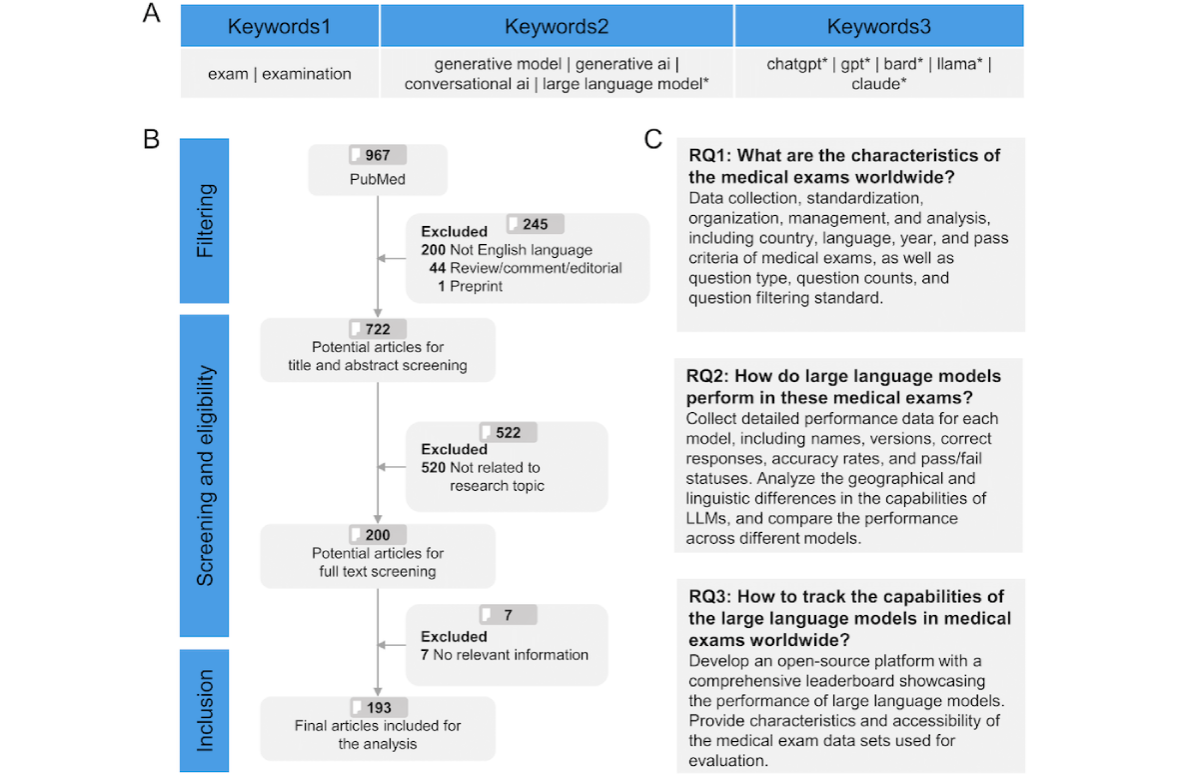
Overview of the study, including (A) the systematic search process for publications related to generative artificial intelligence and large language models (LLMs) on medical exams, (B) article screening and inclusion process used to select studies for analysis, and (C) key research questions addressed by the study. RQ: research question.

In this study, we aimed to address 3 key research questions (RQs) to enhance the understanding and evaluation of LLM capabilities for medical exams conducted worldwide (Figure 1C). These research questions were designed to systematically investigate and document the interplay between LLMs and medical exams, facilitating the advancement of artificial intelligence in medical education.

RQ1 was “What are the characteristics of medical exams worldwide?” This question focused on the collection, standardization, organization, management, and analysis of data pertaining to the medical exams. This detailed characterization will provide an in-depth understanding of the global landscape of medical exams in the evaluation of LLMs.

RQ2 was “How do LLMs perform in these medical exams?” To evaluate the performance of LLMs, we collected extensive performance data for each model, including their names, versions, number of correct responses, accuracy rates, and pass and fail statuses. This analysis included examining geographical and linguistic differences in LLM capabilities and comparing the performance of different models. By doing so, we aimed to identify trends and disparities in LLM performance across various contexts and regions.

RQ3 was “How can the capabilities of LLMs in medical exams worldwide be tracked?” We proposed the development of an open-source platform that features a comprehensive leaderboard to showcase the performance of LLMs on medical exams globally. This platform not only highlights the capabilities of different models but also provides detailed information on the characteristics and accessibility of the medical exam data sets used for evaluation. This platform will serve as a valuable resource for ongoing monitoring and assessment of LLM performance in medical education.

## Methods

### Data Collection

A systematic search was conducted in the PubMed database on April 25, 2024, to identify relevant articles. To ensure comprehensive coverage of the research questions, we selected 3 categories of keywords, as shown in Figure 1A: (1) general terms related to generative artificial intelligence (eg, generative model), (2) specific terms referring to LLMs (eg, ChatGPT), and (3) terms related to medical exams (eg, exam). The full search query used in PubMed was as follows: (generative model[TIAB] OR generative ai[TIAB] OR conversational ai[TIAB] OR large language model*[TIAB] OR chatgpt*[TIAB] OR gpt*[TIAB] OR bard*[TIAB] OR llama*[TIAB] OR claude*[TIAB]) AND (exam[TIAB] OR examination[TIAB]). By leveraging PubMed as the primary data source, we ensured the inclusion of peer-reviewed studies, which provide a high level of evidence quality and reliability. The inclusion criteria encompassed the following: (1) the article described original research, (2) the article was published in the English language, (3) the article investigated the use of at least one LLM on medical exams. The exclusion criteria included the following: (1) regarding article type, reviews, comments, or editorials, as these typically lack primary data and experimental results on LLM performance, which are essential for our analysis, as well as preprints, to ensure all included studies had undergone peer review for accuracy and validation; (2) regarding language, articles published in non-English languages to ensure consistent interpretation of data; (3) regarding content relevance, articles that did not use any LLMs or provide relevant information, such as exam details, model descriptions, and performance evaluation. The inclusion of all candidate articles was verified by 2 researchers. The PRISMA (Preferred Reporting Items for Systematic Reviews and Meta-Analyses) checklist [30] is included in Multimedia Appendix 1. The overall process of article selection is shown in Figure 1B.

### Content Curation

To provide a comprehensive understanding of the capability of LLMs on medical exams worldwide, we manually curated and standardized information for each study, integrating it into a structured database designed for the platform. The curated data informed the key components of the MedExamLLM platform. These data included (1) exam information (name, country or region, language, year, question types, description, and passing criteria for the medical exams), (2) data processing (description of question filters, as well as the number of questions before and after screening), (3) model performance (name of the LLM, number of correctly answered questions, score, accuracy, and whether the platform passed), (4) data availability (whether the data are publicly available and the download link), (5) reference (basic information about the referenced study, including PMID, authors, title, abstract, publication date, publication year, and journal). The detailed descriptions and examples of each item are shown in Table 1.

The search process and curated information were foundational to addressing the 3 RQs: They enable detailed characterization of medical exams (RQ1), support evaluation and comparison of LLM performance (RQ2), and facilitate the creation of a tracking platform for LLM capabilities (RQ3).

During data processing, we followed a multistep protocol to ensure reliability. First, exam details, model names, and performance metrics were extracted and standardized to ensure consistency across studies. This included normalizing exam names, question types, and scoring metrics to align with predefined categories. To mitigate errors, 2 independent researchers (HZ and RW) verified the data entry for accuracy. Any discrepancies were resolved through discussion and, when necessary, by consulting with another researcher (BS) until consensus was achieved. Finally, we used a cross-verification step to ensure data quality and consistency. This involved applying a standardized review of the curated content across all entries, with special attention to aligning these elements with both the original study methodologies and predefined data framework.

The curated information was then integrated into the platform, serving as the backbone for its visualization and interaction capabilities. For instance, standardized exam names and scoring metrics allowed dynamic filtering and comparison of model performance across multiple exams, languages, and geographic regions.

**Table 1:** Information curated for each reference, including exam information, data processing, model performance, data availability, and reference.

Data categories and items		Data description
**Exam information**		
	Exam country	The geographic area or nation state where an exam is conducted
	Exam language	The specific language used in an exam
	Exam year	The year in which an exam was conducted
	Exam name	The name or title of an exam
	Exam description	A detailed description of what an exam covers
	Question type	The category or style of question used in an exam
	Pass criteria	The standards or conditions that must be met for an individual to be considered as having passed an exam
**Data processing**		
	Question filter	A method or process used to select or screen questions for an exam
	Question num before	The number of questions in an exam before a certain filtering process was applied
	Question num after	The number of questions in an exam after a certain filtering process was applied
**Model performance**		
	Model	A type of artificial intelligence, specifically a large language model, designed to simulate human-like interaction by understanding, interpreting, and generating human language
	Correct response	The count of questions answered correctly by the model during a testing
	Score	A numerical representation of the model’s performance on an examination, often calculated based on the total number of correct responses
	Accuracy	A measure of the model’s performance, typically calculated as the ratio of the number of correct responses to the total number of questions
	Pass	Refers to whether a model has met the pass criteria for an exam
Data availability		Refers to whether the data of an exam is accessible or can be downloaded by others for reuse in subsequent research
**Reference**		
	PMID	A unique PubMed identifier assigned to each article in the PubMed database
	Authors	The name list of researchers who conducted the research or published the article
	Title	The heading of the article, which typically summarizes the main subject or focus of the research
	Abstract	The abstract of the article, often including the study’s purpose, methods, results, and conclusions
	Publication date	The specific date on which the article was published
	Publication year	The specific year in which the article was published
	Journal	The name of the academic or scientific journal in which the article was published

### Platform Implementation

The MedExamLLM platform was designed with a focus on accessibility, interactivity, and scalability to support comprehensive benchmarking of LLMs on medical exams. Key design objectives included enabling users to explore performance data across diverse exams and model versions and allowing updates as new LLM results become available. The platform’s architecture integrates a database to manage and structure exam and model data, allowing users to effectively query and visualize performance metrics.

To achieve these aims, we implemented a modular web framework that facilitates data management, interactive data visualization, and user feedback. The platform includes dynamic charting capabilities, enabling users to compare model performance across different exams, languages, countries, and models while providing evidence support from the published literature. These design elements ensure that MedExamLLM can serve as a long-term resource that adapts to evolving needs in medical artificial intelligence research.

### Statistical Analysis

The chi-square test was used to assess the performance differences of LLMs across different languages and model types. The chi-square test was selected for its suitability for comparing categorical variables, enabling us to determine whether observed differences in performance were statistically significant. This approach ensured a rigorous evaluation of both linguistic and model-based performance variability. Statistical analyses were performed using the R software package (version 4.2.1).

### Ethical Considerations

This study exclusively used publicly available data sets and did not involve any experiments on human subjects or animals. Additionally, no private or sensitive patient information was used. Consequently, this research did not require approval from an ethics review board, in accordance with the ethical guidelines of the authors’ institution.

## Results

### Platform Overview

**Figure 2:**
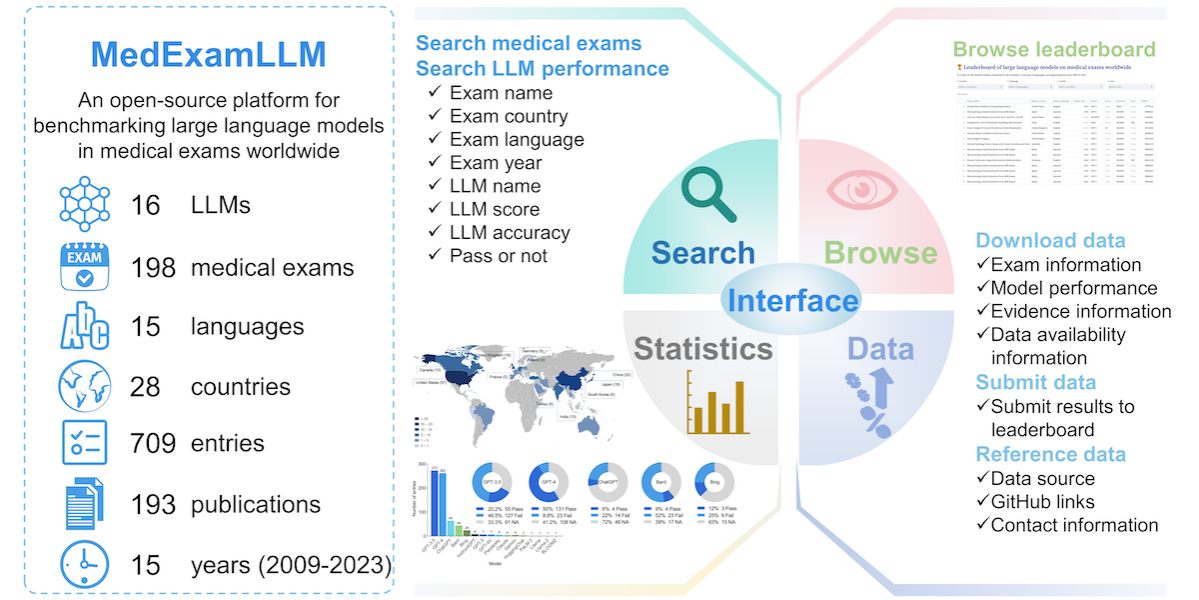
Overview of the structure and features on the MedExamLLM platform, including core modules such as the large language model (LLM) performance leaderboard, medical exam information search, and medical exam data set management, as well as statistics visualization, user submission of new data, and data set download.

The MedExamLLM platform is freely accessible at [31]. It was designed with a user-friendly web interface to facilitate the search, visualization, submission, and download of data. The primary modules include the LLM performance leaderboard module, medical exam information search module, and medical exam data set module (Figure 2).

The LLM performance leaderboard module displays 709 entries, encompassing 16 large models across 198 medical exams from 28 countries and covering 15 languages. The data elements include the standardized names of the medical exams, the countries in which they were administered, the languages used, the year of implementation, the LLM tested, the score of the LLM, the accuracy of the LLM, whether it passed the exam, and related references. The leaderboard can be filtered by country, language, model, and pass status, and it also can be sorted according to the aforementioned data elements.

The medical exam search module presents comprehensive information on the medical exams of interest, including publications, performance, and statistics. The publication information shows all the published articles related to the searched medical exam, including title, author, journal, and year of publication. The performance information includes the model, as well as its score, accuracy, and pass status. The statistical information offers descriptive statistics and visualizations for all LLMs associated with the specific medical exam.

The medical exam data set module provides detailed information on the medical exam data sets, including the data set description, pass criteria, question type, question filtering process, the number of questions before and after filtering, and download methods.

Additionally, the MedExamLLM platform includes submission, download, and statistics modules. The submission module allows users to submit new evaluation results of LLMs on medical exams, which helps to ensure the accuracy, completeness, and timely update of the data in the platform. The download module provides links to all data generated by this study. The statistics module displays comprehensive statistical descriptions and graph visualizations.

### Data Statistics

A total of 967 articles were initially retrieved for consideration. These studies underwent a sequential screening process based on the predefined eligibility criteria. First, the metadata of the studies were examined, leading to the exclusion of 200 articles written in non-English languages; 44 articles categorized as a review, comment, or editorial; and 1 article identified as a preprint. Next, of the remaining 722 articles, the titles and abstracts were manually assessed. Among these, 520 articles were found to be irrelevant to the research topic and were excluded. Subsequently, a thorough examination of the full texts of the remaining articles was conducted, resulting in the identification of 7 articles that did not provide relevant information. Finally, a total of 193 articles were included in this study (Figure 1B).

The types and versions of LLMs used, as well as the categories and years of medical exams, varied across different articles. For example, Cheong et al [32] investigated the performance of 3 models (GPT-3.5, GPT-4, and Google Bard) across 10 categories of the Sleep Medicine Certification Board Exam. This research involved a total of 30 experiments, resulting in the inclusion of 30 entries on the MedExamLLM platform. Zong et al [17] evaluated the performance of ChatGPT on the Chinese National Medical Licensing Examination, Chinese National Pharmacist Licensing Examination, and Chinese National Nurse Licensing Examination from the year 2017 to the year 2021. A total of 15 experiments were conducted, leading to the inclusion of 15 entries on the MedExamLLM platform. At the time the manuscript was written, the MedExamLLM platform comprised 709 structured entries curated from 193 scholarly articles.

### Distribution of Countries, Languages, and Years for Medical Exams

**Figure 3:**
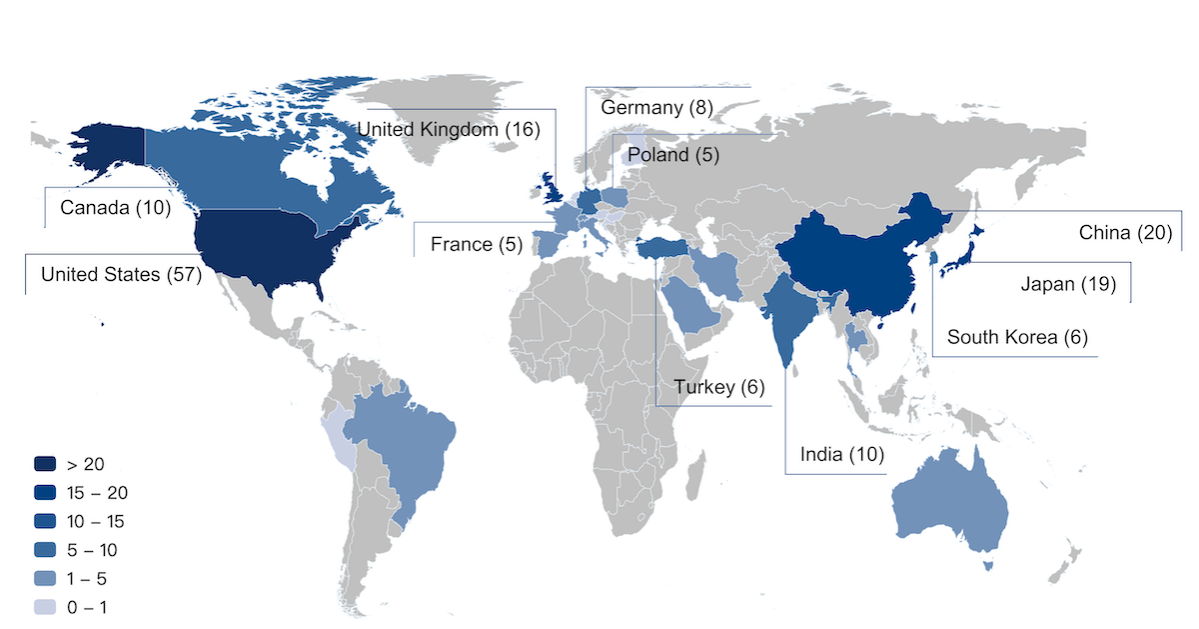
World distribution of the medical exams in the MedExamLLM platform. The map was created using ECharts.

The MedExamLLM platform collected medical exams in 15 languages from 28 countries, spanning from the year 2009 to the year 2023. The United States had the highest number of medical exams, with 57 publications, followed by China (20 publications), Japan (19 publications), the United Kingdom (16 publications), Canada (10 publications), India (10 publications), Germany (8 publications), Turkey (6 publications), South Korea (6 publications), France (5 publications), and Poland (5 publications; Figure 3). English emerged as the predominant language used in these medical exams, with 127 publications using exams conducted in English, followed by Chinese (20 publications), Japanese (19 publications), German (5 publications), Korean (5 publications), and Polish (5 publications; Table 2). The majority of the studies used medical exams administered in the year 2022 (72 publications), followed by 2023 (35 publications), 2021 (23 publications), 2020 (16 publications), and 2019 (11 publications; Table 2).

The sources of the exam questions were diverse. Most studies collected questions from national medical licensing examinations from different countries. Some studies also used questions sourced from examination books, question banks, medical textbooks, and websites. These exams encompassed various hospital roles, including physicians, surgeons, pharmacists, nurses, and other health care professionals. They also covered a wide range of specialties and disciplines, including urology, radiology, cardiology, neurology, ophthalmology, anesthesiology, dermatology, plastic surgery, neurological surgery, and gynecology and obstetrics.

**Table 2:** Distribution of publications by exam language and exam year in the MedExamLLM platform.

Exam characteristics			Publications, n
**Language**			
	English	127	
	Chinese	20	
	Japanese	19	
	German	5	
	Korean	5	
	Polish	5	
	French	3	
	Spanish	3	
	Portuguese	3	
	Hebrew	2	
	Indian	2	
	Italian	2	
	Dutch	1	
	Turkish	1	
	Peruvian	1	
**Year**			
	2009	1	
	2010	1	
	2011	1	
	2012	1	
	2013	2	
	2014	3	
	2015	4	
	2016	5	
	2017	6	
	2018	8	
	2019	11	
	2020	16	
	2021	23	
	2022	72	
	2023	35	

### Differences in LLMs’ Capabilities Across Geographic and Linguistic Contexts

**Figure 4:**
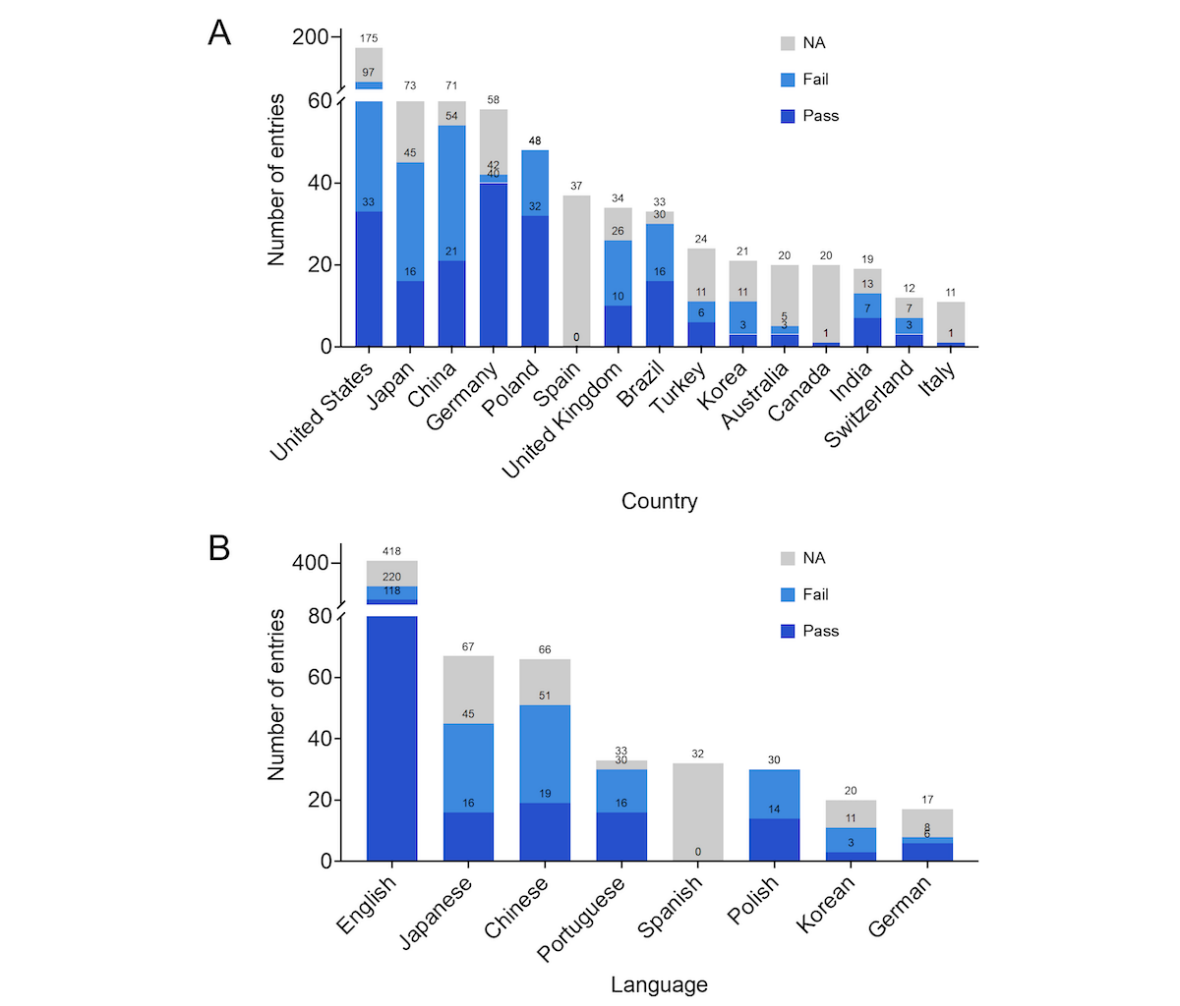
Capabilities of large language models across geographic and linguistic contexts, as indicated by the pass rates on medical exams across (A) 15 countries and (B) 8 languages. NA: not applicable.

We further analyzed the pass rates of LLMs on medical exams across different countries and languages. The pass rate was defined as the ratio of exams passed by a model to the total number of evaluative entries, excluding those that did not report a pass or fail outcome. We evaluated 15 countries with more than 10 entries each. As shown in Figure 4A, the United States had the most entries, totaling 175, of which 33 showed that the model passed the exam, 64 showed that the model failed, and 78 did not indicate whether the model passed or failed, resulting in a pass rate of 34% (33/97). The numbers of entries in Japan and China were 73 and 71, respectively, and the pass rates were 36% (16/45) and 39% (21/54), respectively. The highest pass rate was in Germany, at 95% (40/42), followed by Poland, with a pass rate of 67% (32/48). Most of the entries from Canada and Italy did not indicate whether the LLMs passed or failed. For languages, we evaluated 8 languages with more than 10 entries each. As shown in Figure 4B, English had the most entries, totaling 418, of which 118 indicated that the model passed the medical exam, 102 indicated that the model failed, and 198 records did not specify whether the model passed or failed, resulting in the highest pass rate of 53.6% (118/220). German had the highest pass rate at 75% (6/8), but it is important to note that the number of entries was not large. The pass rates for other languages were 53% (16/30) for Portuguese, 47% (14/30) for Polish, 37% (19/51) for Chinese, 36% (16/45) for Japanese, and 27% (3/11) for Korean. Spanish had a total of 32 entries, but none indicated whether the model passed the exam.

Our chi-square analysis revealed significant pass rate differences among languages. English was the predominant language for these exams; however, models demonstrated varying performance when other languages were evaluated. For instance, the pass rate difference between English and Chinese exams was statistically significant (*P*=.02), as was the difference between English and Japanese exams (*P*=.049), while the differences between English exams and exams in Portuguese (*P≥*.99), Polish (*P*=.65), Korean (*P*=.18), and German (*P*=.39) were not statistically significant. These results reveal significant differences in the capabilities of LLMs across different geographical and linguistic backgrounds.

### Performance and Pass Rates of LLMs

The MedExamLLM platform currently encompasses a total of 16 LLMs. As illustrated in Table 3, these models are ranked based on their frequency of use. The GPT series models are extensively used, with GPT-3.5 being the most frequently used, with a total of 273 entries, followed closely by GPT-4 with 262 entries and ChatGPT with 64 entries. Bard and Bing were used 44 and 24 times, respectively. Whether the LLM can pass the medical exams is a crucial aspect highlighted in the MedExamLLM platform. In the MedExamLLM platform, 28.2% (200/709) of entries indicated that the LLM successfully passed the medical exams, while 29.1% (206/709) indicated a failure to pass. Of the entries, 42.7% (303/709) lacked a clear statement regarding whether the model passed the medical exam. Among the top 5 most frequently used models, the GPT-4 model showcased a significantly higher pass rate than the other models. Remarkably, 50% (131/262) of the entries indicated that GPT-4 successfully passed the medical exam, while only 8.8% (23/262) indicated that it failed and 41.22% did not indicate whether it passed (Table 4). Additionally, the chi-square analysis revealed that GPT-4 outperformed other LLMs, with significant differences in pass rates compared with GPT-3.5 (*P*<.001), ChatGPT (*P*<.001), Bard (*P*<.001), and Bing (*P*<.001). These results underscore the variability in LLM effectiveness across different model types, highlighting the need for model-specific considerations when applying LLMs to medical education.

Table 5 shows examples of national licensing examinations from different countries, as well as the performance of LLMs on those exams. For instance, the GPT-3.5 model achieved an accuracy of 54.67% on the 2021 Chinese National Medical Licensing Examination, which consists of 600 questions in Chinese [17]. The GPT-4V model achieved an accuracy of 72% on the 2023 Japanese National Medical Licensing Examination, which consists of 108 questions in Japanese [26]. ChatGPT achieved accuracies of 45.4%, 54.1%, and 61.5% for Steps 1, 2CK, and 3, respectively, on the 2022 United States Medical Licensing Examination, which consists of 350 questions in English [22].

**Table 3:** Frequency of the use of various large language models (LLMs) across medical exams.

LLM	Frequency of use, n
GPT^a^-3.5	273
GPT-4	262
ChatGPT	64
Bard	44
Bing	24
InstructGPT	8
GPT-3	7
GPT-4V	7
Perplexity	6
Claude	4
Gemini	4
HuggingChat	2
PaLM 2	1
Llama	1
Llama-2	1
BLOOMZ	1

^a^GPT: generative pretrained transformer.

**Table 4:** Pass rates of the top 5 most frequently used large language models (LLMs).

LLM	Passed, n (%)	Failed, n (%)	Not reported, n (%)
GPT^a^-3.5 (n=273)	55 (20.2)	127 (46.5)	91 (33.3)
GPT-4 (n=262)	131 (50)	23 (8.8)	108 (41.2)
ChatGPT (n=64)	4 (6)	14 (22)	46 (72)
Bard (n=44)	4 (9)	23 (52)	17 (39)
Bing (n=24)	3 (12)	6 (25)	15 (63)

^a^GPT: generative pretrained transformer.

**Table 5:** Examples of national medical licensing examinations from different countries and the related performance of large language models.

Exam	Country	Language	Year	Questions, n	Model	Accuracy, %	PMID
Australian Medical Council Licensing Examination	Australia	English	N/A^a^	150	GPT^b^-4	79.3	37528548
Chinese National Medical Licensing Examination	China	Chinese	2021	600	GPT-3.5	54.67	38355517
French Medical Licensing Examination	France	French	N/A	300	ChatGPT	22	37553555
German State Examination in Medicine	Germany	German	2022	252	GPT-3.5	66.7	37530052
Iranian Medical Licensing Examination	Iran	English	2023	200	GPT-4	68.5	38081765
Italian Medical Licensing Examination	Italy	Italian	N/A	300	ChatGPT	73	37553555
Japanese National Medical Licensing Examination	Japan	Japanese	2023	108	GPT-4V	72	38470459
Korean National Licensing Examination for Korean Medicine Doctors	South Korea	Korean	2022	340	GPT-4	66.18	38100393
Peruvian National Licensing Medical Examination	Peru	Peruvian	2023	180	GPT-4	86.67	37981579
Saudi Medical Licensing Examination	Saudi Arabia	English	N/A	220	GPT-4	88.6	37829968
United States Medical Licensing Examination	United States	English	2022	350	ChatGPT	45.4, 54.1, and 61.5 for USMLE^c^ Steps 1, 2CK^d^, and 3, respectively	36812645

^a^N/A: not applicable.

^b^GPT: generative pretrained transformer.

^c^USMLE: United States Medical Licensing Examination.

^d^CK: clinical knowledge.

## Discussion

### Principal Findings

The MedExamLLM is a comprehensive compilation of the latest research of LLMs on medical exams worldwide, including data from 198 medical exams across 28 countries in 15 languages from 2009 to 2023, and the evaluation performance of 16 LLMs on these medical exams. This study showed that the United States dominates in the number of medical exams and publications, with English being the primary exam language. The GPT series models, especially GPT-4, excelled on these exams, with significantly higher pass rates compared with other models. Additionally, this study revealed significant variability in the capabilities of LLMs across different geographic and linguistic contexts, as well as the strengths and weaknesses of various models on different medical exams. MedExamLLM serves as a valuable resource for educators, researchers, and developers in the fields of clinical medicine and artificial intelligence.

### Expansion Beyond Previous Research

This study expands and deepens the research in several aspects. First, most previous studies focused on medical exams in a single country or a specific language, lacking a global perspective. The MedExamLLM platform fills this gap by providing comprehensive data across a wide range of countries and languages. Second, previous studies often focused only on the performance of a specific model or a few models, whereas this study systematically evaluated the performance of 16 different LLMs on various medical exams, providing more extensive and diverse evidence. Third, previous research usually focused on static exam results, while this study further compares the dynamic performance of these models across different years, countries, and languages. This expansion of the temporal and spatial dimensions enhances the depth and breadth of the study and provides an important foundation for future research and applications. Finally, this study constructs an open-source, freely accessible, and publicly available online platform that provides comprehensive performance evaluation information and evidence-based knowledge of LLMs on worldwide medical exams.

This platform not only offers a valuable data resource for researchers but also provides evidence for educators and policymakers. Through detailed performance evaluations and comparisons, educators can better understand the advantages and limitations of LLMs, enabling more effective application of these technologies in classroom teaching. Meanwhile, researchers can use this platform to further optimize, enhance, and improve the capabilities of LLMs in medical education applications [33].

### Potential of and Challenges With LLMs in Medical Education

MedExamLLM systematically compares the performance of LLMs on medical exams and reveals the considerable potential of artificial intelligence technologies to improve the quality and efficiency of medical education. The application of LLMs in the field of medical exams is expected to enhance students’ exam preparation efficiency and improve their exam scores [34]. In the future, with the continuous evolution and advancements of LLM technology, its application in medical education will become more widespread. However, it is important to note that, although LLMs can provide valuable assistance, they are unlikely to replace individual roles in medical education and training [35]. These models can serve as useful tools to help teachers prepare classroom teaching materials and help students strengthen their understanding of medical knowledge. Furthermore, the use of LLMs for medical education should be combined with traditional learning methods and conducted under the guidance of qualified medical professionals [36,37].

MedExamLLM offers practical applications for educators seeking to integrate artificial intelligence into curriculum development and student assessment. The platform records the performance for a variety of LLMs and evidence across different regions, languages, and medical specialties, providing valuable insights for educators to select the model best suited to their specific context. For example, teachers can use MedExamLLM to identify models that perform optimally in their language or region, helping them tailor their instructional strategies. In a previous study [16], we illustrated how LLMs like ChatGPT effectively assist with enhancing students’ academic writing quality and facilitating teachers’ grading processes. This aligns with MedExamLLM’s broader objective of equipping educators with reliable AI-based insights to improve both student learning outcomes and assessment efficiency.

### Ethical Considerations

MedExamLLM systematically evaluates the performance of LLMs across a wide array of medical exams, showcasing the potential benefits and critical considerations of artificial intelligence integration into medical education. Although our platform highlights the promising utility of LLMs to aid with exam preparation and broaden access to medical knowledge, several ethical implications are important for stakeholders to consider, particularly regarding data privacy, model bias, and the risks associated with artificial intelligence errors in high-stakes settings.

First, data privacy is crucial given that medical exams often contain sensitive information about exam content, structure, and performance trends. Medical institutions and artificial intelligence developers must ensure that data privacy is safeguarded, especially if LLMs are trained on specific medical exam data sets. A recent study suggested that underrepresentation across languages ​​and regions in data sets may lead to challenges with deploying LLMs in clinical [38].

Second, model bias presents a significant ethical challenge. Since LLMs learn from diverse data sets, any inherent biases can result in unequal model performance across different demographic and linguistic groups. In MedExamLLM, we observed variability in LLM pass rates across exams from different countries and languages, suggesting potential biases in how well models perform in various contexts. This variability underscores the need for ongoing efforts to improve the representativeness of training data and incorporate fairness metrics when assessing model outcomes.

Finally, the use of LLMs on medical exams involves the potential risk of artificial intelligence errors, which, in a medical context, can lead to serious consequences if misapplied or misunderstood. For instance, LLMs may incorrectly answer clinically relevant questions or provide misleading explanations, which could impact the educational experiences of students relying on these models. In addition, although studies have suggested that LLMs can generate medical exam questions, direct use also increases the risk of misinformation [39-42]. MedExamLLM’s analysis of LLM accuracy and pass thresholds offers insights into the models’ reliability, but this is not a replacement for human verification in medical education. Educators and institutions should use these tools as a supplement to traditional learning methods, ensuring that LLMs are applied within a structured framework that includes expert validation.

### Future Road Map

The MedExamLLM platform is designed with a forward-looking approach to accommodate the evolving landscape of LLMs in medical education. Our plans for the future development of MedExamLLM include 4 aspects.

The first is regular platform updates and expansion of model evaluations. MedExamLLM will incorporate periodic updates to include performance data for newer LLMs as they become available. To address the rapid advancements in LLM technology, the platform will be updated annually, allowing for timely additions of emerging models and versions. This will provide users with the latest comparative performance data across diverse exam contexts. This initial version focuses on data from PubMed, and the platform is designed to evolve, with future updates planned to incorporate additional data sources and expand coverage. The second is user feedback. A feedback mechanism has been integrated into the platform to allow users, including educators and researchers, to suggest improvements and submit their own performance data. This submission module enables users to contribute new evaluation results from additional exams or model assessments, enhancing the platform’s data completeness and scope. Feedback from users will also inform updates to ensure the platform remains user-centered and relevant to the needs of the medical education and artificial intelligence communities. The third is broadening the scope to include diverse exam formats and expanded language coverage. We plan to expand MedExamLLM’s coverage by incorporating different exam formats, such as case-based questions, to provide a more comprehensive view of LLMs’ capabilities with various medical evaluation formats. Additionally, efforts will be made to include exam data sets in less commonly represented languages to improve the linguistic diversity in our platform. The fourth is practical applications and real-world impact. In the future, we will focus on evaluating LLMs’ effectiveness in real-world educational settings beyond exams. As described in our previous publication [16], by exploring the utility of LLMs in simulated clinical scenarios and interactive teaching contexts, we aim to broaden the understanding of these models' capabilities and limitations in medical education.

### Limitations

This study has some limitations. First, the data mainly come from publicly available peer-reviewed journal publications. However, many studies are initially published on preprint platforms, which may introduce bias in data acquisition and completeness. The exam data from some countries and languages may be incomplete or not detailed enough, thus affecting the representativeness of the results. Second, LLMs are frequently updated, with new models and versions continuously emerging, which presents a unique challenge for a platform aiming to provide up-to-date evaluations. To address this, we implemented a submission module in MedExamLLM that enables users to contribute new results for emerging LLMs. The platform is designed to undergo periodic updates, allowing for ongoing comparative analysis as models evolve. Finally, the study focuses on LLM performance on medical exams and does not explore real-world applications of these models in medical education. Although performance on exams can offer insights into some aspects of artificial intelligence proficiency, it remains inadequate for capturing the complete range of capabilities exhibited in real-world scenarios. Therefore, future research needs to evaluate the effectiveness of LLMs in educational settings to better understand their strengths and limitations.

### Conclusion

We described the development and functionality of MedExamLLM, an open-source, freely accessible, and publicly available online platform that provides comprehensive performance evaluation information and evidence-based knowledge of LLMs on medical exams around the world. The MedExamLLM platform comprises data from 198 medical exams conducted in 28 countries, covering 15 languages, and spanning from 2009 to 2023. MedExamLLM reveals variations in LLM performance across different countries and languages, as well as the strengths and weaknesses of different models for various medical exams. By contributing to the growing knowledge on LLM capabilities for medical exams, MedExamLLM highlights both the potential benefits and limitations of their use in medical education. Furthermore, it provides valuable insights for the integration of artificial intelligence technologies in medical education, making it a valuable resource for educators, researchers, and developers in the fields of clinical medicine and artificial intelligence.

## Appendix

Multimedia Appendix 1 PRISMA (Preferred Reporting Items for Systematic Reviews and Meta-Analyses) checklist.

## Abbreviations

GPT: generative pretrained transformer
LLM: large language model
PRISMA: Preferred Reporting Items for Systematic Reviews and Meta-Analyses
RQ: research question

## Data Availability

The data analyzed and reported in this study are available at [31].
